# Evaluation of ferroptosis-based anti-leukemic activities of ZnO nanoparticles synthesized by a green route against Pre-B acute lymphoblastic leukemia cells (Nalm-6 and REH)

**DOI:** 10.1016/j.heliyon.2024.e36608

**Published:** 2024-08-22

**Authors:** Muhammad Hossein Ashoub, Mahnaz Amiri, Ahmad Fatemi, Alireza Farsinejad

**Affiliations:** a Stem Cells and Regenerative Medicine Innovation Center, Kerman University of Medical Sciences, Kerman, Iran; b Student Research Committee, Faculty of Allied Medicine, Kerman University of Medical Sciences, Kerman, Iran; c Neuroscience Research Center, Institute of Neuropharmacology, Kerman University of Medical Science, Kerman, Iran; d Cellular and Molecular Research Center, Gerash University of Medical Sciences, Gerash, Iran; e Department of Hematology and Medical Laboratory Sciences, Faculty of Allied Medicine, Kerman University of Medical Sciences, Kerman, Iran

**Keywords:** Anti-leukemic activities, Ferroptosis, Green chemistry, Zinc oxide nanoparticles

## Abstract

**Background:**

Our research presents an efficient and practical method for producing Zinc Oxide nanoparticles (ZnO NPs), which have anti-leukemic effects based on ferroptosis.

**Methods:**

The *black cardamom* extract was employed as a capping and reducing agent for the green synthesis. The NPs have been characterized via scanning electron microscopy, X-ray diffraction, and Fourier-transform infrared spectroscopy. Additionally, leukemic and normal cells were exposed to ZnO NPs (25, 50, 75, 100, 150, 200, and 300 μg/mL) for 24 and 48 h. The cell vitality was then measured using the MTT test. Moreover, ferroptosis indicators were assessed via commercial testing kits, and finally, qRT-PCR and flow cytometry were used to measure gene expression and cell death.

**Results:**

The findings displayed that green synthesized ZnO NPs reduced the survival of leukemic cells, with IC50 values of 150.89 μg/ml for Nalm-6 and 101.31 μg/ml for REH cells after 48 h. The ZnO NPs increased ferroptosis by significantly increasing MDA, intracellular iron, *ACSL4*, *ALOX15*, and *p5*3 mRNA expressions while significantly decreasing GSH and GPx activity levels and *SLC7A11* and *GPx4* mRNA expressions. On the other hand, ZnO NPs exhibited no toxicity toward normal cells.

**Conclusions:**

The research suggests that ZnO NPs synthesized using the green approach can induce ferroptosis in leukemic cells by disrupting redox homeostasis and increasing intracellular iron levels, potentially enhancing the benefits of anti-leukemic therapies in the future.

## Graphical abstract

**Figure:**
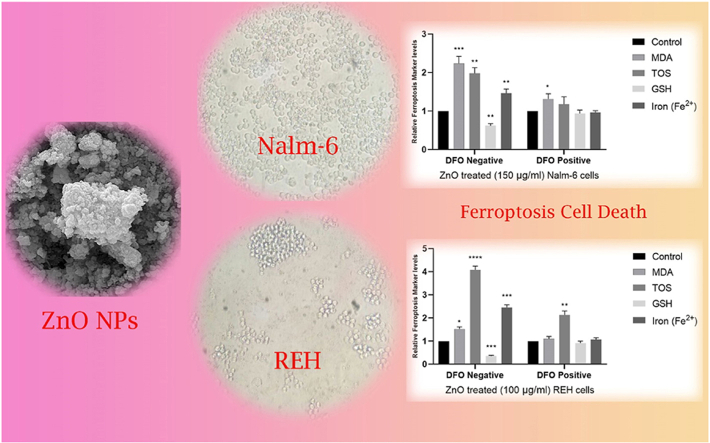

## 1 Introduction

A hematopoietic stem cell malignant clone causes leukemia, a liquid tumor that severely impairs the hematopoietic system's ability to operate normally. The unchecked proliferation of immature B-cell or T-cell lineage cells characterizes the lymphoid cell cancer known as acute lymphoblastic leukemia (ALL). Even though ALL in children can be treated in up to 90 % of cases, relapses happen in about 20 % of patients [1,2]. Additionally, because of resistance to chemotherapy and radiation treatments, some patients do not respond to treatment and are at a significant risk of relapsing. Due to the low specificity and stability of conventional treatment regimens, it is urgently necessary to look for novel and combinational treatment approaches for leukemia [3,4].

Ferroptosis differs from apoptosis, autophagy, and necrosis in the context of morphological, genetic, and biochemical traits. Ferroptosis, a process involving lipid reactive oxygen species (ROS) accumulation, is influenced by iron [5]. Specific cancer cells can decrease their vulnerability to ferroptosis by suppressing the ferroptosis pathway, which leads to resistance against treatments [5]. However, triggering ferroptosis can re-sensitize these cells to conventional therapies [6]. In the case of leukemia, ferroptosis significantly regulates various leukemia pathologies, including acute lymphoblastic leukemia (ALL) [7,8]. Dysregulation of iron metabolism and excess iron buildup are linked to leukemia, mainly due to iron's pro-oxidative properties and harmful effects on DNA [9]. Therefore, Ferroptosis shows potential as an intervention in the fight against leukemia. As a result, one of the research hotspots on this topic is stimulating ferroptosis in leukemia cells, which looks to be a valuable method for leukemia therapy [10].

Nanotechnology is rapidly being used to treat leukemia because of its ability to administer anti-leukemic medications more efficiently and selectively [11,12]. Additionally, for some medical purposes, such as tumor targeting and cancer cell elimination, nanoparticles (NPs) can act as therapeutic agents or carriers [13,14]. Most NP-based treatments have been focused on treating solid tumors, whereas less attention has been paid to non-solid malignancies, including lymphoma and leukemia. Nanoparticle study has been driven by the need for new technological applications in biological sciences, medicine delivery, and therapy [15,16]. Nanotechnology advancements have sparked interest in developing multifunctional NPs that can induce ferroptotic processes, potentially for cancer treatment, with most studies utilizing iron or iron oxide NPs [17]. Therefore, studying different types of NPs can enhance our comprehension of the underlying mechanisms and aid in engineering more efficient and functional nanosystems.

Zinc Oxide (ZnO) NPs are frequently employed in antibacterial treatments for skin protection and food additives for nutritional reasons. Additionally, ZnO NPs have been engaged in a number of medicinal settings, yet despite this, ZnO NPs are among the poisonous metal oxide NPs, and different manufacturing techniques result in ZnO NPs with various characteristics [18,19]. Numerous academic studies have displayed that ZnO NPs are also harmful due to producing reactive oxygen species (ROS). Additionally, measurements have been made of the relationship between ZnO physicochemical parameters and toxicity [20]. Chemical reduction is the primary method for synthesizing metal NPs among various available methods [21]. Plant extracts are frequently used to synthesize NPs due to their cost-effectiveness, stability, minimal contamination risk, and ease of preparation, making them an attractive tool in the scientific domain [22]. However, the chemical reduction method by using chemical materials may result in the adsorption of toxic chemicals, which requires additional steps to remove them. This may adversely affect medical applications [23,24]. Consequently, it is imperative to create a strategy for producing ecologically safe NPs [25,26]. ZnO NPs produced through eco-friendly, green, and biological methods offer numerous benefits. These NPs have been employed as nanocatalysts in energy generation and have demonstrated antimicrobial properties against various microorganisms. Moreover, they have exhibited significant efficacy in photocatalytic activities and hold promise for applications in anticancer treatments [27].

*Black cardamom*, Amomum subulatum, is a herbaceous plant in the Zingiberaceae family that has been shown to cure various diseases, including coughs, lung congestion, discomfort, and stomach disturbances. Modern phytoanalytical and pharmacological techniques are broadening their therapeutic applications, with bioactive chemicals from plant component extracts connected to antioxidant, antibacterial, analgesic, anti-inflammatory, anti-ulcer, cardio-adaptogen, and hypolipidemic activities [28]. *Black cardamom* contains abundant essential oils, colors, proteins, and minerals. Black cardamom is high in potassium and contains trace quantities of magnesium [29,30]. Recent research has demonstrated that *black cardamom* can be used medicinally in cancer therapy, with one study indicating its cytotoxic impact on lung cancer cells [31]. Furthermore, using *Black cardamom* extract as a reducing agent for green synthesis is warranted due to its rapid reducing capacity [32].

Therefore, the novelty of current work lies in a green and sustainable method for synthesizing ZnO NPs, aligning with our previous research [33] and the global environmental concerns and offering a cost-effective, scalable solution and then, the green-synthesized ZnO NPs are applied to induce ferroptosis in leukemia cells, exploring a new avenue for anti-leukemic therapies. The study investigates the impacts of ZnO NPs on Nalm-6 and REH leukemia cells, enhancing understanding of cell-specific responses and paving the way for personalized leukemia treatment strategies.

## 2 Material and methods

### 2.1 Materials

*Black cardamom* is freely accessible in markets, and Zn (NO_3_)_2_.5H_2_O was purchased from Merck Company and hasn't undergone any additional purification before usage. Penicillin-streptomycin, fetal bovine serum (FBS), RPMI 1640, phosphate-buffered saline (PBS), Deferoxamine (DFO, D9533), and ZVAD-FMK (V116) were obtained from Sigma Aldrich. The iron assay kit (cat. no. ab83366) was purchased from Abcam. Ferroptosis and antioxidant assay kits were obtained from Navand Salamat, Iran. The Iranian Arshanzist Youtab Company (Kerman, Iran) donated all other materials.

### 2.2 Black cardamom extract preparation

*Black cardamom* has been employed as a reducing and capping agent in the synthesis technique described here. To make an aqueous extract, 10 g of *black cardamom* was immersed in 100 ml of distilled water and heated at 100 °C for 1 h. After boiling, Whatman filter sheets were used to filter the obtained extract. The filtered extract was stored at 4 °C for later use.

### 2.3 Coprecipitation synthesis of ZnO NPs

At room temperature, 1 mM of Zn (NO_3_)_2_.5H_2_O solution was added to diluted *black cardamom* extract to synthesize NPs. The mixture was stirred for 2 h at room temperature using a magnet stirrer. Then 0.1 M NaOH was added to reach a pH of 12. The obtaining of NPs was indicated by a change in color in the reaction mixtures. Following synthesis, the NPs were separated by centrifugation at 16,000 rpm for 10 min, cleaned with sterile water, and extensively washed with 80 % ethanol. The end output was a white color powder substance that was dried overnight in an oven at 60 °C.

### 2.4 Characterizations

The X-ray diffraction (XRD) pattern recorded by a Philips-X'pertpro, an X-ray diffractometer using Ni-filtered Cu Ka radiation, and Fourier transforms infrared (FT-IR) spectra recorded in KBr pellets on a Nicolet Magna-550 spectrometer were among the technologies used to identify the ZnO NPs. Scanning electron microscopy (SEM) was also conducted using the TESCAN MIRA 3 device.

### 2.5 Cell culture

Cell lines for acute lymphoblastic leukemia (Nalm-6 and REH) were acquired from Tehran, Iran's Pasteur Institute collection. The Pasteur Institute has an extensive record with cell line authentication, and the catalogs of the cell lines are available on their website: https://en.pasteur.ac.ir/Department-of-Cell-Bank. Furthermore, we have consistently obtained cell lines from the Pasteur Institute and published multiple studies [[34], [35], [36], [37]]. Cell lines were grown in RPMI-1640 with l-glutamine, FBS, and antibiotics in a humidified environment. Peripheral blood mononuclear cells (PBMCs) were extracted from healthy donors using Ficoll-Hypaque density gradient and centrifugation. Written informed consent was obtained from all participants. The pellet was resuspended and cultivated in 6 mL of complete media under the same conditions as Nalm-6 and REH cells.

### 2.6 MTT assay

The toxicity of ZnO NPs was assessed using an MTT colorimetric assay. Hence, 1 × 10^4^ cells were seeded in 96-well plates with various concentrations of ZnO NPs (25, 50, 75, 100, 150, 200, and 300 μg/mL) for 24 and 48h with or without other co-treatments with inhibitors. The cells were treated with 100 μL of MTT solution at 37 °C for 4 h. After solubilization, optical absorbance was measured at 570 nm with an enzyme-linked immunosorbent assay reader. The proportion of metabolic activity in treated cells was determined vs untreated cells. Each sample was assayed in triplicate, and the experiment was repeated 3 times. ZnO NPs were suspended in RPMI 1640 medium and sonicated for 10 min at 40 W to prevent agglomeration before cell treatment. The concentrations of ZnO NPs utilized in our research were determined based on prior studies [33,38]. Also, 24- and 48-h exposure times are frequently utilized in biological and chemical studies. These durations are selected due to their ability to allow discernible effects to manifest while also being feasible within laboratory settings.

### 2.7 Annexin V-FITC/PI staining analysis

The study utilized flow cytometry to assess the effect of ZnO NPs on apoptosis in cells. 4 × 10^5^ cells were seeded into six-well plates and treated with different doses of ZnO NPs. After 48 h, the cells were collected and analyzed using the Annexin-V Apoptosis Detection Kit, and the results were analyzed using FlowJo.7.6.1 software. Each sample was assayed in triplicate, and the experiment was repeated 3 times.

### 2.8 Total oxidant status (TOS), total antioxidant capacity (TAC), and malondialdehyde (MDA) measurement

The total oxidant status of a sample is calculated to assess the cumulative effects of oxidants like ROS and reactive nitrogen species (RNS), which cause significant harm to cellular contents [39]. Total non-enzymatic antioxidant capacity analysis demonstrates a cell's ability to protect itself from oxidative stress-related damage. The Total Antioxidant Capacity Assay Kit examines the levels of a mixture of antioxidants or antioxidants alone. Lipid peroxidation is a valuable biomarker of oxidative stress. The production of malondialdehyde results from oxidative stress on polyunsaturated lipids that are frequently triggered by reactive oxygen species. 5 × 10^5^ cells were put into each well of a six-well plate. Following 48 h of treatment with ZnO NPs (IC50 values), cells were collected and counted, and supernatants were used for analysis. MDA levels, TAC, and TOS were determined using commercial assays and following the manufacturer's instructions. Each sample was assayed in triplicate, and the experiment was repeated 3 times.

### 2.9 The measurement of reduced glutathione (GSH) and glutathione peroxidase (GPx) activity

The total reduced glutathione, commonly called glutamyl-cysteinyl-glycine, is a crucial antioxidant in many species. In addition, GSH plays a critical role in diseases. Assessing intracellular GSH indicates a cell's general health and ability to survive harmful stimuli and ferroptosis [40]. In the glutathione peroxidase test method, glutathione peroxidase oxidizes GSH to produce Glutathione disulfide (GSSG) to reduce cumene hydroperoxide. Glutathione reductase (GR) degrades NADPH while decreasing the GSSG to make GSH. As a result, NADPH (measured at OD = 340 nm) declines in terms of GPx activity. 4 × 10^5^ cells were put into each well of a 6-well plate. Following 48 h of treatment with ZnO NPs (IC50 values), cells were collected and counted, and supernatants were used for analysis. According to the manufacturer's recommendations, glutathione GPx activity and GSH were measured via commercial assay kits. Each sample was assayed in triplicate, and the experiment was repeated 3 times.

### 2.10 Iron determination assay

To quantify intracellular ferrous iron (Fe^2+^) levels, the iron assay kit was applied following the manufacturer's instructions. The experiment was carried out three times for each group. Each sample was assayed in triplicate, and the experiment was repeated 3 times.

### 2.11 RNA isolation and preparation of cDNA

After the treatment of ZnO NPs (IC50 values), the cells were harvested at the designated times, and the total RNA was extracted using YTzol Pure RNA, and its quality and purity were evaluated using a NanoDrop 1000 Spectrophotometer. The RevertAid First Strand cDNA Synthesis kit performed the reverse transcription reaction according to the manufacturer's instructions.

### 2.12 Quantitative real-time PCR

The study used quantitative real-time PCR to analyze changes in mRNA expression of genes. The reaction involved 7.5 μL of Real Q Plus 2x Master Mix Green, 1.5 μL of cDNA product, 1 μL of forward and reverse primers, and 4 μL of nuclease-free water. The Rotor-Gene Q Real-time PCR System was used for thermal cycling, with initial activation at 95 °C for 15 min and 40 cycles, including denaturation and annealing/elongation. The specificity of the products was confirmed through a melting curve analysis. After accounting for the β-actin reference gene, the fold change was estimated relative to the control using the comparative Ct (2^−ΔΔCT^) method. Each sample was assayed in triplicate, and the experiment was repeated 3 times. Table 1 contains the real-time PCR primers.

**Table 1:** Primer sets for quantitative real-time polymerase chain reaction.

primers	Forward	TM	Reverse	TM
ALOX15	5′-CTGGCCGACCTCGCTATCAA-3′	62.02	5′-CTTCCTTCCAGGAGTCCCGC-3′	62.25
ACSL4	5′-CGAGCTTTCCGAGTGCCAGG-3′	63.44	5′-CAGTACAGCCAAGGCAGTTCAA-3′	61.07
p53	5′-GAGCGCTTCGAGATGTTCCG-3′	61.48	5′-ATGGCGGGAGGTAGACTGAC-3′	61.04
GPX4	5′-GCCTTTGCCGCCTACTGAAG-3′	61.65	5′-ACGAAGCCCCGGTACTTGTC-3′	62.16
SLC7A11	5′-GCTCCATGAACGGTGGTGTG-3′	61.58	5′-AGAGGAGTGTGCTTGCGGAC-3′	62.44
β-actin	5′-CCAACCGCGAGAAGATGA-3′	57.09	5′-TCCATCACGATGCCAGTG-3′	57.06

### 2.13 Statistical analysis

The study utilized SPSS version 20.0 to compare mean values among groups, reporting experimental data as mean standard deviation (SD). Each test was carried out three times. First, a two-way ANOVA test was performed to calculate the statistical analysis, and post hoc Tukey was then utilized to determine significance. Significant differences in results were determined to exist at *P < 0.05.

## 3 Results and discussion

### 3.1 Characterization of ZnO NPs

#### 3.1.1 XRD analysis

**Fig. 1:**
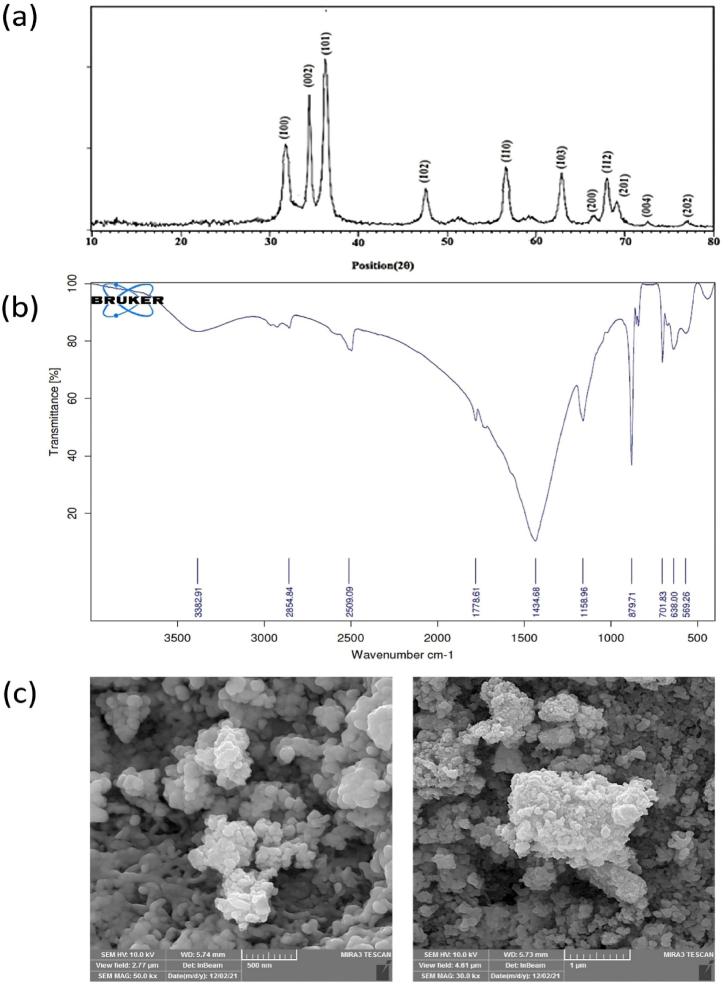
**Characterization of nanoparticles. (a)** XRD patterns of ZnO NPs. **(b)** FTIR patterns of ZnO NPs. **(c)** SEM images of ZnO NPs at different magnifications.

Using XRD analysis, the structural characteristics of the final product were reported. In Fig. 1a, the acquired XRD pattern for the samples of ZnO NPs made using the coprecipitation technique was displayed. Peaks identified in the wurtzite ZnO (JCPDS no. 42–1451) that are indexed at (100), (002), (101), (102), (110), (103), (200), (112), (201), (004), and (202) support the crystal structure of ZnO NPs [41,42]. The NPs were crystalline and well-arranged. The absence of impurity peaks demonstrates the material's ideal purity. Also, the average size of crystallite samples was determined to be 22 ± 1.23 nm. Using Debye Scherer's formula, the size was calculated [43].

#### 3.1.2 FTIR analysis

In real-world applications, FTIR spectroscopy gives information about the functional groups in the analyzed sample [44]. Under the coprecipitation approach, FTIR analyses were made for a range of 4000–400 cm^−1^ and displayed in Fig. 1b. The ions' oscillation in the crystal lattice causes the bands in this range [45]. The interaction of functional groups in the *Black cardamom* extract with ZnO NPs could be accountable for the observed alterations in the sample spectrum's peak positions [32]. It's crucial to highlight that the electrons donated by the extract's functional groups could lead to a reduction in zinc ions. Moreover, the extract's negative functional groups might exhibit a stabilizing effect [46]. The biomolecules from the extract appear to be capped or attached to the surface of the ZnO NP. This is indicated by the slight shift and minor changes in various related peaks and intensities in the FT-IR spectrum of the biosynthesized ZnO NPs. The absorption bands in the FTIR spectra of ZnO NPs at 3382.91 cm^−1^ are attributed to the stretching vibrations of the O–H groups in water, alcohol, and phenols [47]. The jagged peaks at 2854.84 cm^−1^ are associated with C–H stretching, and the peaks at 2509.09 cm^−1^ could be due to vibrations of residual organic extract in the ZnO NPs [48]. The band at 1778.61 cm^−1^ is due to the C=O stretch of polyphenols. The protein in the extract contributes to the C–N stretch at the 1434.68 cm^−1^ band, and the C–O stretching results in the 1158.96 cm^−1^ band. The C–O–C stretch vibrations peak at 879.71 cm^−1,^ and the peaks at 638 and 701.83 cm^−1^ are due to C–H bending vibrations [49]. Additional peaks at 569.26 cm^−1^ in the IR spectrum of the ZnO NPs may be characteristic peaks of ZnO molecules [50]. The FT-IR analysis of green-produced ZnO-NPs revealed that the Zn–O absorption band has been found at 400–600 cm^−1^ wavelengths, which is consistent with our observations [50,51]. The IR spectra of the prepared ZnO NPs demonstrate the active participation of these biomolecules in the reduction and stabilization of the ZnO NPs. The coordination of ZnO NPs with –OH and C=O groups could be responsible for the stabilization and capping of the synthesized ZnO NPs [52].

#### 3.1.3 SEM analysis

Pure NPs that were generated using the coprecipitation approach were investigated using SEM; various magnifications of the pictures are shown in Fig. 1c. In the micrographs, the presence of agglomerates is evident, and the primary particles present a "spheroidal" morphology. It was found that nanocrystalline powders were randomly dispersed in terms of their sizes and shapes. Also, the average size of particles was 30 ± 5 nm. The smaller crystallite size than the primary nanoparticle size suggests polycrystalline NPs, possibly due to multiple crystallites or amorphous regions [53]. However, the close values of the primary nanoparticle size and crystallite size suggest nearly single-crystalline nanoparticles or crystallites as the dominant feature [54]. Thus, the ZnO NPs likely comprise one or a few crystallites.

### 3.2 Cellular studies

#### 3.2.1 ZnO NPs trigger ferroptosis in leukemic cells

**Fig. 2:**
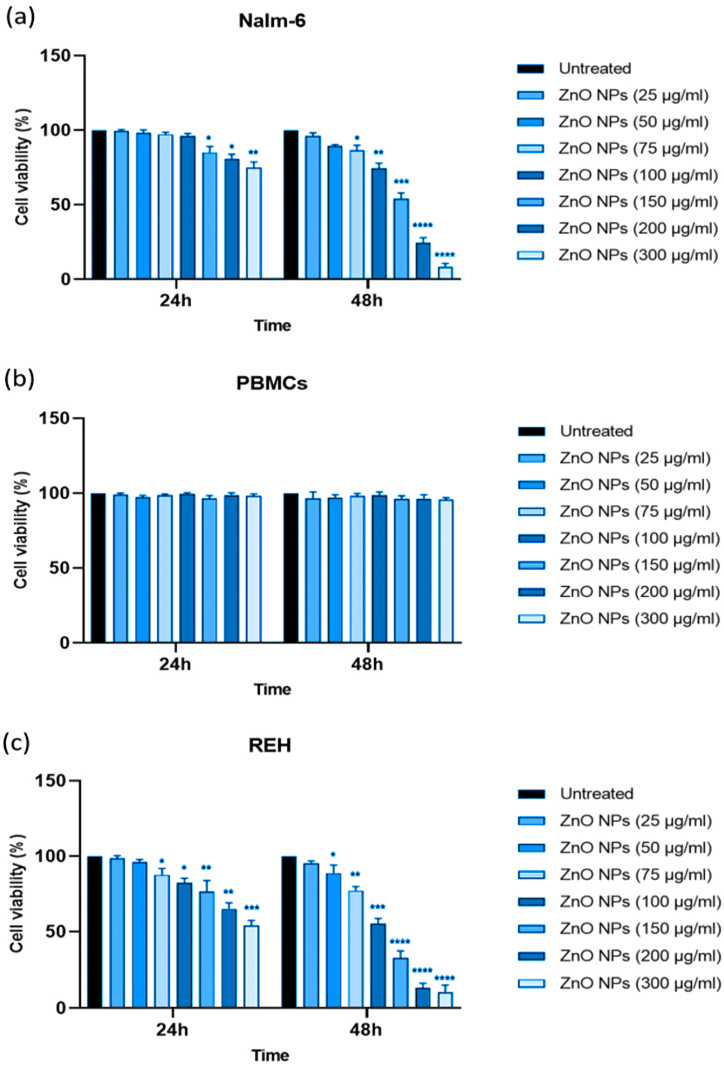
**Treatment with ZnO NPs reduced the viability of Nalm-6 and REH cells. (a)** Nalm-6, **(b)** PBMCs, and **(c)** REH cells treated with ZnO NPs at 25, 50, 75, 100, 150, 200 and 300 μg/ml for 24 h and 48 h. The MTT assay was used to assess cell viability. Each data point is expressed as mean ± SEM of 3 independent tests. *P < 0.05, **P < 0.01, ***P < 0.001, ****P < 0.0001. SEM, standard error of the mean.

**Fig. 3:**
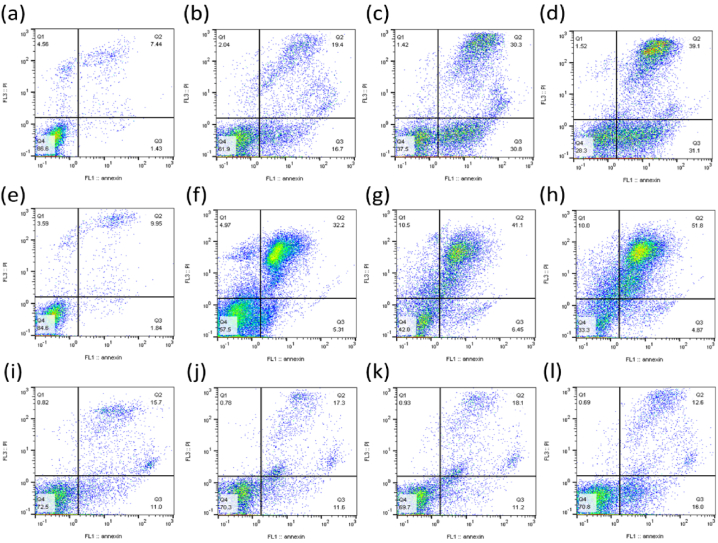
**ZnO NP**^**'**^**s induction of cell death in Nalm-6 and REH cells. (a)** Untreated Nalm-6 cells, **(b)** Nalm-6 cells treated with 150 μg/ml of ZnO NPs, **(c)** Nalm-6 cells treated with 200 μg/ml of ZnO NPs, **(d)** Nalm-6 cells treated with 300 μg/ml of ZnO NPs, **(e)** Untreated REH cells, **(f)** REH cells treated with 100 μg/ml of ZnO NPs, **(g)** REH cells treated with 150 μg/ml of ZnO NPs, **(h)** REH cells treated with 200 μg/ml of ZnO NPs, **(i)** Untreated PBMCs, **(j)** PBMCs treated with 50 μg/ml of ZnO NPs, **(k)** PBMCs treated with 150 μg/ml of ZnO NPs, **(l)** PBMCs with 300 μg/ml of ZnO NPs for 48 h. Then, flow cytometry analyzed cells for Annexin-V and Annexin-V plus Propidium Iodide (PI). Annexin V-propidium iodide (PI) staining by flow cytometry was used to assess cell death. Q1, Q2, Q3, and Q4 indicate PI-positive, Annexin-V/PI double-positive, Annexin-V positive, and Annexin-V/PI double-negative cells, respectively. One representative experiment of 3 performed is shown.

**Fig. 4:**
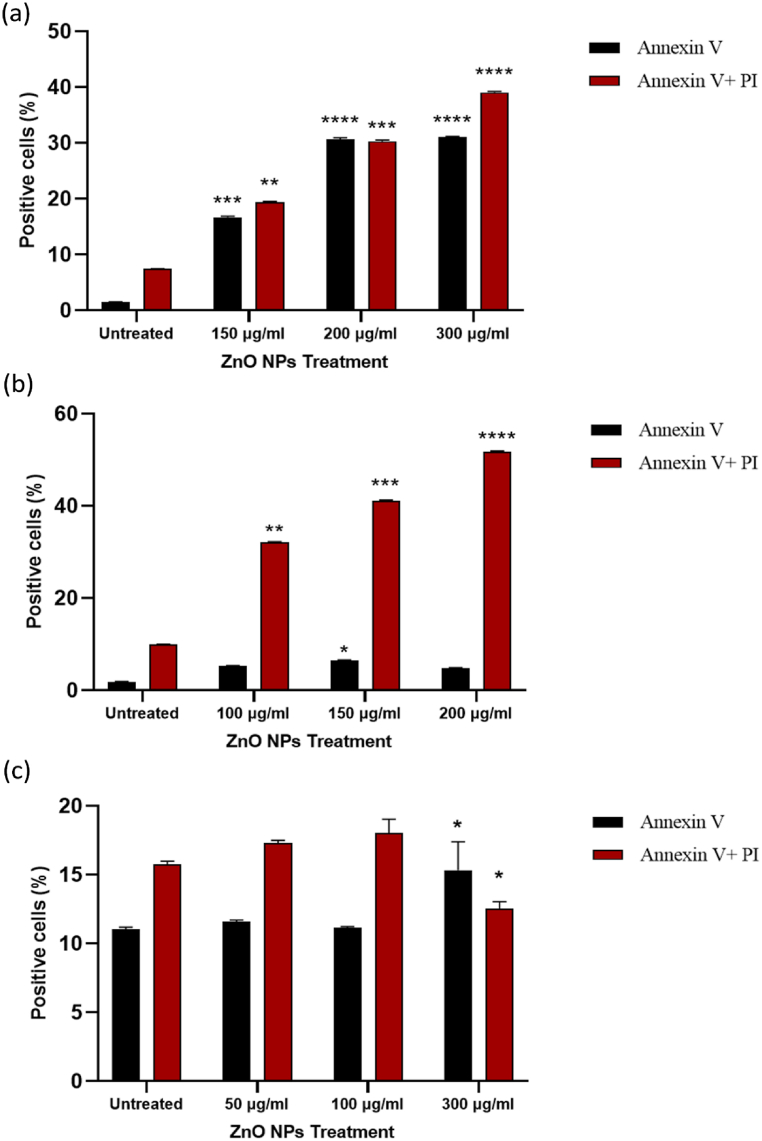
**ZnO NP's effect on the quantity of Annexin-V/PI double-positive and Annexin-V positive cells. (a)** Nalm-6 cells treated with ZnO NPs at 0, 150, 200, and 300 μg/ml for 48 h, **(b)** REH cells treated with ZnO NPs at 0, 100, 150, and 200 μg/ml for 48 h, and **(c)** PBMCs cells treated with ZnO NPs at 0, 50, 150, and 300 μg/ml for 48 h *P < 0.05, **P < 0.01, ***P < 0.001, ****P < 0.0001.

**Fig. 5:**
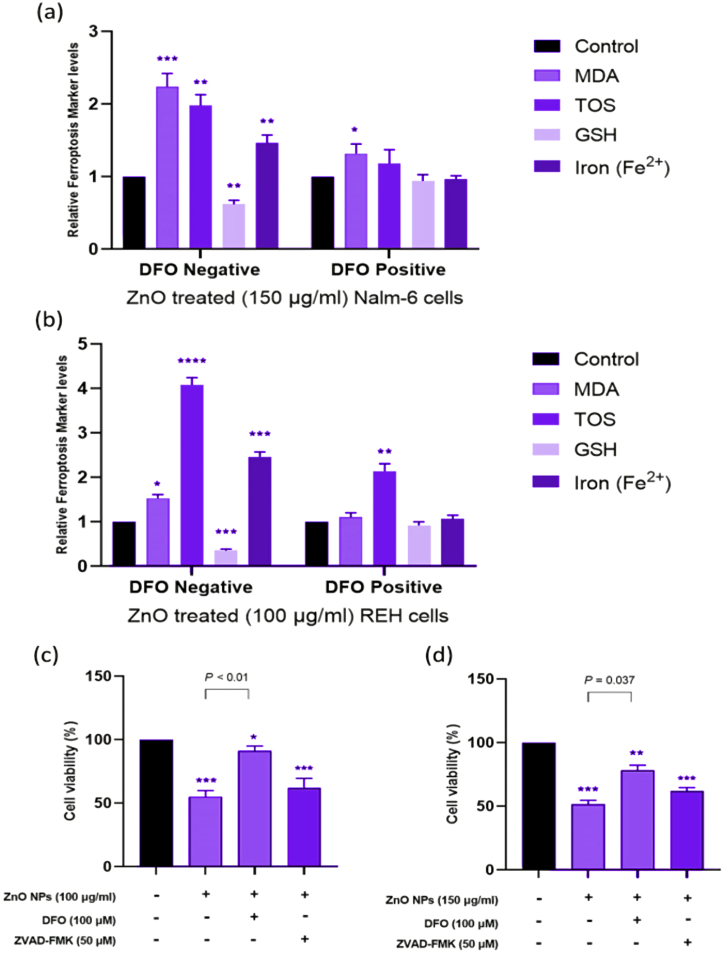
**DFO pretreatment eliminates the effect of ZnO NPs on cell viability and ferroptosis markers.** Nalm-6 **(a, c)** and REH **(b, d)** cells were preincubated with various inhibitors, including ZVAD-FMK (50 μM) and DFO (100 μM) for 2 h, followed by ZnO NPs treatment for 48 h. Each data point is expressed as mean ± SEM of 3 independent tests. *P < 0.05, **P < 0.01, ***P < 0.001, ****P < 0.0001. MDA, malondialdehyde; TOS, Total Oxidant Status; GSH, Reduced Glutathione; TAC, Total Antioxidant Capacity; GPx, Glutathione Peroxidase.

**Fig. 6:**
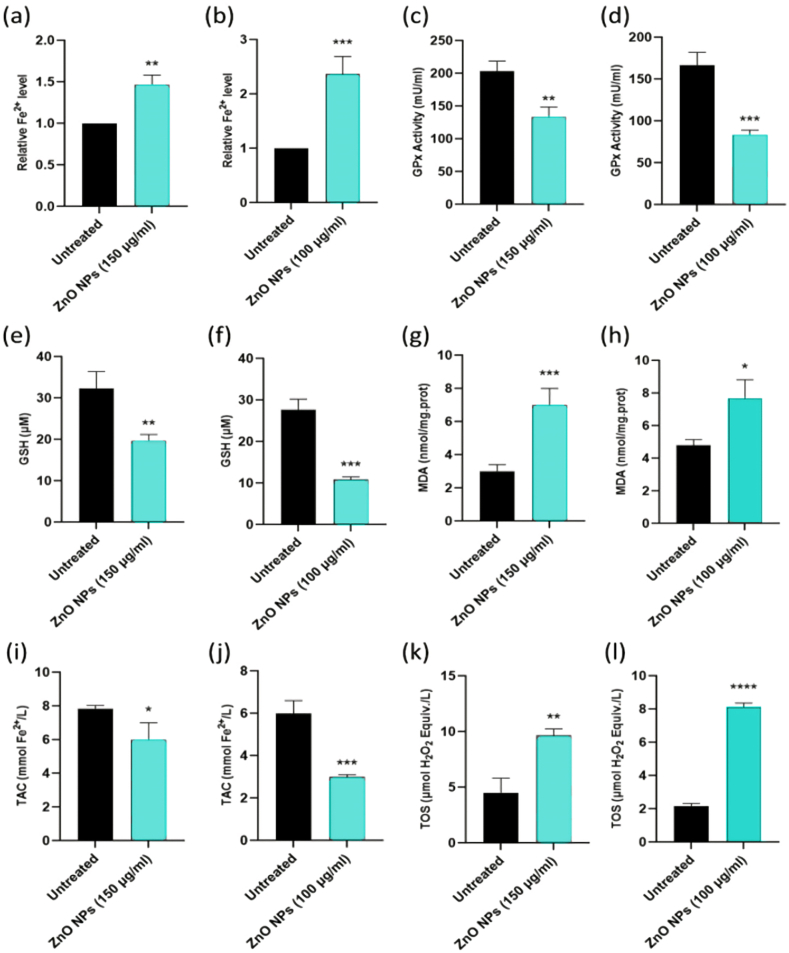
**ZnO NPs treatment induces cell ferroptosis in Nalm-6 and REH cells via upregulation of oxidative stress, lipid peroxidation, iron accumulation, and impairment of antioxidant defense.** The ferroptosis markers in Nalm-6 and REH cells followed by ZnO NPs (150 μg/ml for Nalm-6 and 100 μg/ml for REH) treatment for 48 h was examined. The intracellular Fe^2+^ levels in the Nalm-6 **(a)** and REH **(b)** cell lines were evaluated by an iron assay kit. GPx activity in the Nalm-6 **(c)** and REH **(d)** cell lines was assessed via a GPx activity commercial kit. GSH levels in the Nalm-6 **(e)** and REH **(f)** cell lines were evaluated via a GSH commercial kit. The MDA accumulation in the Nalm-6 **(g)** and REH **(h)** cell lines was evaluated by a lipid peroxidation assay kit. TAC levels in the Nalm-6 **(i)** and REH **(j)** cell lines were evaluated by a Total Antioxidant Capacity assay kit. TOS levels in the Nalm-6 **(k)** and REH **(l)** cell lines were evaluated by a Total Oxidant Status assay kit. Each data point is expressed as mean ± SEM of 3 independent tests. *P < 0.05, **P < 0.01, ***P < 0.001, ****P < 0.0001. MDA, malondialdehyde; TOS, Total Oxidant Status; GSH, Reduced Glutathione; TAC, Total Antioxidant Capacity; GPx, Glutathione Peroxidase.

We chose Nalm-6 and REH cell lines due to their established use in pre-B acute lymphoblastic leukemia research and their connection to ALL patients, providing a relevant biological context for studying nanoparticles' effects on this cancer type and the difference between these cell lines may be due to their genetic and phenotypic characteristics [55,56]. These differences might influence the cells' responses to ZnO NPs, providing a more comprehensive understanding of the nanoparticles' effects [57]. Following treatment with varying dosages of green synthesized ZnO NPs, the survival of Nalm-6 (Fig. 2a), REH (Fig. 2c), and PBMCs (Fig. 2b) was assessed via the MTT test at 24 and 48 h. The findings displayed that ZnO NPs reduced the survival of Nalm-6 and REH cells, with corresponding IC50 values of 150.89 μg/ml and 101.31 μg/ml after 48 h, respectively. ZnO NPs had no discernible impacts on the viability of PBMCs. Following treatment with ZnO NPs for 48 h in Nalm-6 and REH cell lines, annexin V-FITC/PI staining findings showed a dose-dependent elevation in annexin V-FITC/PI-positive cells (Fig. 3(a–h) and Fig. 4a and b). To confirm the safety of ZnO NPs, the PBMC cells were incubated (48 h) with ZnO NPs. As presented in Fig. 3(i–l) and Fig. 4c, PBMCs exposure with ZnO NPs did not elevate the annexin V-FITC/PI-positive cells (a little elevation in annexin V-FITC positive cells was seen, but since annexin V-FITC/PI-positive cells were decreased compare to the untreated cell the overall cell death was similar to untreated cells). The caspase inhibitor Z-VAD-FMK was pre-treated to cells to ascertain the type of cell death. As presented in Fig. 5c and d, MTT studies proved that Z-VAD-FMK did not eliminate the toxicity caused by ZnO NPs in both Nalm-6 and REH cell lines. In contrast, ferroptosis inhibitor DFO enhanced cell survival in Nalm-6 (*P* < 0.01) and REH cells (*P* = 0.037) to offset the effects of ZnO NPs (Fig. 5c and d). Thus, the ferroptosis inhibitor significantly reduced the induced cell death, leading to the conclusion that the cell death was due to ferroptosis. To confirm this observation, biomarkers of ferroptosis were further examined. Ferroptosis is characterized by elevated oxidative stress, lipid peroxidation, and iron accumulation, which can deteriorate the cell's antioxidant defense system [58]. Our findings showed that after 48 h of treatment, ZnO NPs significantly lowered the levels of total antioxidant capacity, GSH depletion, and GPx activity and raised TOS, intracellular iron levels (Fe^2+^), and MDA levels in Nalm-6 and REH cell lines (Fig. 6(a–l)). All of these findings indicated an increase in ROS and ferroptosis induction. Additionally, an elevation in intracellular iron, a crucial component in the induction of ferroptosis, was also observed. To investigate the impacts of the ferroptosis inhibitor on these markers, the cells were once again co-treated with DFO. It was observed that the inhibitor mitigated the impact of the NPs on the cells, reducing the induced elevated intracellular iron. Also, MDA and TOS levels that were elevated by ZnO NPs were suppressed in the presence of DFO in Nalm-6 and REH cells (Fig. 5a and b). These findings, taken together, offer strong proof that leukemia cells underwent ferroptosis following ZnO NPs exposure. Also, the variations in response to ZnO NPs between REH and Nalm-6 cell lines can be attributed to various factors, including cell line characteristics such as metabolic activities, growth rates, and genetic makeup. These characteristics can influence how cells interact with and respond to external substances like ZnO NPs. The extent of ROS generation also varies depending on these cell lines' characteristics, and the rate of NPs dissolution is based on factors like the pH of the cellular environment, which contribute to the observed differences [59,60].

Research has previously displayed that ZnO NPs can cause toxicity in endothelial cells through ferroptosis, a process characterized by increased intracellular iron concentrations, lipid peroxidation, and cell death. The effects depend on the dosage and duration of exposure. However, ferrostatin-1 and deferiprone, lipid reactive oxygen species scavengers, can mitigate ZnONPs-induced cell death [61]. A study found that ZnO NPs trigger ferroptosis in mice's cerebral cortex and PC-12 cells. The source of the cytotoxicity is the interaction between zinc ions and iron within the organism. ZnO NPs increase zinc ion concentration in the brain, potentially inducing iron-dependent cell death. This highlights the complex relationship between zinc and iron in ZnONPs-induced cytotoxicity [62].

Several theories have been proposed on why ZnO NPs have a cytotoxic impact on malignant cells. Research shows that virus-like silica NPs coated with ZnO can reduce hydrogen sulfide (H_2_S) content in colorectal cancer cells, inhibiting their proliferation. This also suppresses tumors through ferroptosis activation. NPs' low toxicity and high safety have been confirmed through toxicological and pathological analyses. A study on tumor tissue models showed a selective increase in ferroptosis-associated proteins and a decrease in GPX4 expression, confirming its potential in colorectal cancer treatment. These findings support previous cellular experiments [63]. ZnO NPs have been found to reduce breast cancer cell resistance to Doxorubicin (Dox). ZnO NPs suppressed the expression of the ABCC9 gene, reducing cell survival. They also decreased GSH levels and increased MDA, ROS, and iron levels in BC/Dox cells stimulated with erastin. These findings suggest that ZnO NPs induce ferroptosis and reduce Dox resistance in breast cancer, highlighting their potential therapeutic value [64]. Synthesized ZnO nanoparticles may impact cancer cells through reactive oxygen species production, oxidative stress, free radical effects, physical interaction with membranes, and increased intracellular Zn^2+^ ion concentrations. Therefore, ZnO NPs are an intriguing choice for cancer theragnostic due to their biocompatibility, simple manufacturing processes, and remarkable capabilities [65].

The standard mechanism that causes ferroptosis is an overabundance of ROS, which is notable given the various characteristics of NPs. After internalization, NPs go positively to mitochondria and disrupt the antioxidant defense mechanism. As an alternative, NPs may naturally have a proinflammatory impact that might produce ROS. Whatever their composition or other qualities, most NPs employ these two methods. Iron-oxide-based NPs for anticancer treatment have been created using the Fenton process and Haber-Weiss reaction [66]. Iron accumulation is a common effect of ZnO NPs exposure, even though ZnO NPs complicatedly regulate genes linked to iron absorption, storage, and export. This suggests that the mechanism of iron dysregulation is crucial in forming ROS caused by ZnO NPs [67,68]. Alternately, ROS-mediated mitochondrial damage may impair the formation of iron-sulfur cluster proteins, leading to iron accumulation in mitochondria [69,70]. Transition metal ions' abnormal homeostasis, such as Fe^2+^/Fe^3+^, Zn^2+^, and Ca^2+^, is crucial in the etiology of many illnesses. For example, an investigation emphasized the relationship between ROS signaling and Zn^2+^ homeostasis, indicating their dependency. These metal ions are present in dynamic pools in mitochondria and are combined with the appropriate metalloproteins [71].

#### 3.2.2 Elevation of ferroptosis-related molecular pathways in ZnO NPs-treated cells

**Fig. 7:**
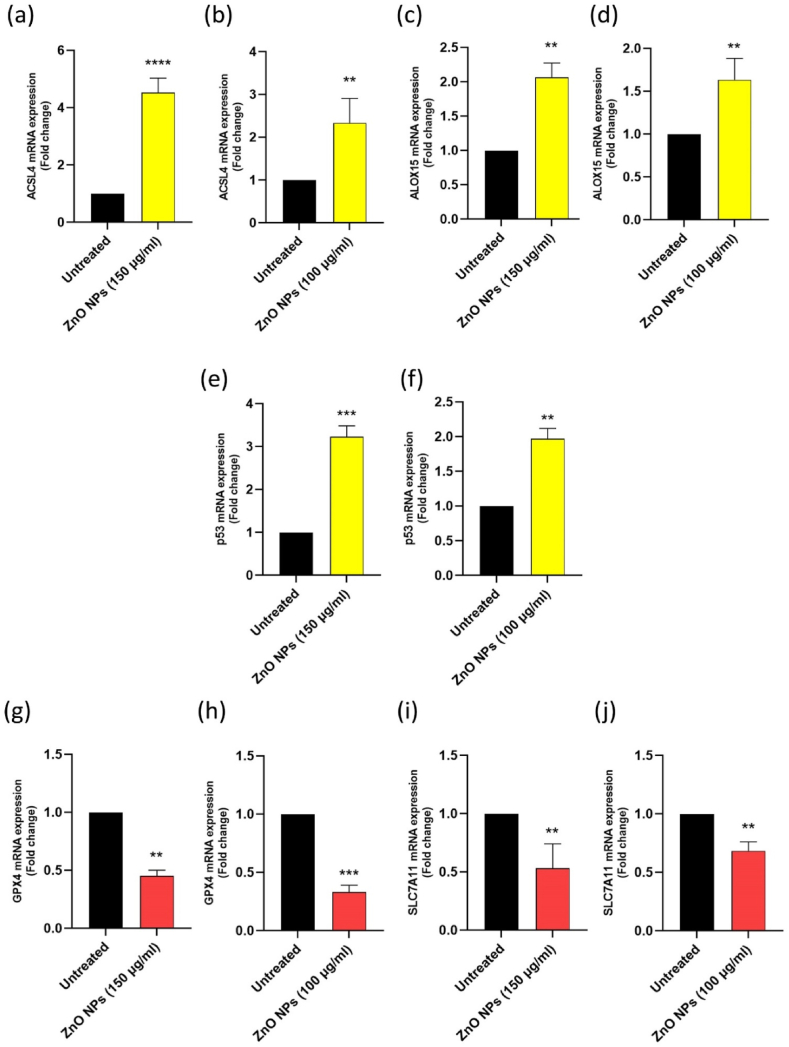
**ZnO NP**^**'**^**s effect on the mRNA expression of ferroptosis-related genes in Nalm-6 and REH cells.** qRT-PCR was performed to examine the ACSL4 **(a)**, ALOX15 **(c)**, p53 **(e)**, GPx4 **(g)**, and SLC7A11 **(i)** mRNA expression levels in Nalm-6 cells after ZnO NPs (150 μg/ml) treatment for 48 h. The ACSL4 **(b)**, ALOX15 **(d)**, p53 **(f)**, GPx4 **(h)**, and SLC7A11 **(j)** mRNA expression levels in REH cells after ZnO NPs (100 μg/ml) treatment for 48 h. Each data point is expressed as mean ± SEM of 3 independent tests. *P < 0.05, **P < 0.01, ***P < 0.001, ****P < 0.0001. ACSL4, Acyl-CoA Synthetase Long-Chain Family Member 4; GPx4, Glutathione Peroxidase 4; SLC7A11, Solute Carrier Family 7 Member 11. ALOX15, Arachidonate 15-Lipoxygenase.

In our study, we used a 48-h time point to evaluate the impact of ZnO NPs. This decision was informed by the observed decrease in cell viability at 48 h, which was more pronounced than at 24 h, as evidenced by the MTT test results. Consequently, we calculated the IC50 values at this time point and proceeded with further experimental testing using these IC50 values and a 48-h timeframe. Selecting an appropriate time point is crucial for analyzing molecular and biochemical changes in response to treatment [72]. We can simultaneously correlate observed phenotypic effects with underlying alterations by examining gene expression and biochemical changes. This approach enhances the chance of detecting alterations and transcriptional events related to treatment effects, facilitating better comparability with other studies and contributing to a more comprehensive understanding of the treatment's impact on cells. Thus, we assessed whether ZnO NPs cause lipid peroxidation by assessing the levels of *GPx4* and *SLC7A11*. ZnO NPs lowered the amounts of *GPx4* and *SLC7A1*1 mRNA in both Nalm-6 (*P* < 0.01) (Fig. 7g–i) and REH cells (*P* < 0.01, and *P* < 0.001, respectively) (Fig. 7h–j). Deleting the ACSL4 gene led to ferroptosis resistance because the acyl-CoA synthetase long-chain family member 4 (ACSL4) aids in the buildup of lipid intermediates during ferroptosis. Alternatively, lipid hydroperoxide production mediated by lipoxygenase (LOX) promotes ferroptosis. Consequently, we used qRT-PCR to test the *ACSL4* and *ALOX15* genes, and the findings showed that both genes' expression levels had increased in Nalm-6 (*P* < 0.0001, and *P* < 0.01, respectively) (Fig. 7a–c) and REH cells (*P* < 0.01) (Fig. 7b–d). *Jiang* et al.*'s* groundbreaking discovery suggested that p53 sensitizes cells to ferroptosis [73].

Additionally, oxidative stress was thought to be linked to the activation of p53. It's interesting to note that ZnO NPs have been linked to many reports of p53 activation [74,75]. Results from qRT-PCR demonstrated that 48 h after treatment, ZnO NPs enhanced *p5*3 mRNA expression in Nalm-6 (*P* < 0.001) and REH cells (*P* < 0.01) (Fig. 7e and f). Cysteine imports and GSH levels both decrease because of P53's binding to the SLC7A11, a component of the cystine/glutamate antiporter [76]. In our study, the *SLC7A1*1 mRNA levels were significantly reduced in the NP-treated groups than in the control groups (*P* < 0.01) (Fig. 7i and j). Notably, p53 alone cannot cause ferroptosis despite mounting evidence supporting the action of p53 in the regulation of ferroptosis.

Interestingly, depending on the experimental conditions, zinc has been shown to prevent or speed up apoptosis in various investigations. Extreme ROS levels trigger apoptosis through p53, while low ROS circumstances encourage the restoration of oxidative balance. Notably, ROS generation may be increased due to p53 activation by controlling specific genes. The role of p53 in cell death caused by ZnO NPs is unclear, as cell deaths share the ROS/p53 axis. p53 is involved in apoptosis, necrosis, and autophagy, but its essential role in cell death remains unknown [77,78]. This suggests that p53 can be a ferroptosis regulator but interacts with other factors and pathways. In some instances, loss of p53 can sensitize cells to ferroptosis. For example, in colorectal cancer cells, p53 loss restricts DPP4 nuclear localization and increases lipid peroxidation, facilitating ferroptosis [79]. Therefore, p53 is not the sole contributor to ferroptosis but rather one of the contributors to its observed effects. Our prior project on the toxicity of ZnO NPs synthesized via the hydrothermal procedure towards acute promyelocytic leukemia cells displayed that ZnO NPs significantly elevated the *ACSL4* and *p53*. Simultaneously, they markedly reduced the mRNA levels of *SLC7A11* and *GPx4*. These alterations collectively accelerated the process of ferroptotic cell death. This shows that ZnO NPs could be leveraged as a beneficial agent in treating acute promyelocytic leukemia [33].

The application of NPs has significantly elevated in recent years. However, the majority of these NPs damage both cancerous and healthy cells. The successful bio-synthesis of ZnO NPs, exhibiting a variety of shapes and sizes, has been achieved using different sections of medicinal plants. This process involves a model approach where divalent Zinc ions (Zn^2+^) from the metal salt undergo a reaction with the bioactive constituents in the plant extract. Typically, the phytochemicals' aromatic hydroxyl (OH) groups bond with Zn^2+^ ions, resulting in a stable complex. This complex subsequently decomposes upon exposure to elevated temperatures, producing ZnO NPs. This innovative procedure presents a promising avenue for the ecologically friendly production of ZnO NPs [80]. In addition, ZnO nanobiohybrids, synthesized using plant extracts, could offer several advantages over traditional ZnO NPs in biological applications. Their unique properties could be beneficial in cancer treatment [81,82].

## 4 Conclusion

This research concentrates on synthesizing ZnO NPs, which are synthesized in the presence of *black cardamom* extract as a caping and alkalizing agent via the coprecipitation method. The spheroidal green synthesized NPs indicated approximately 30 nm size. The present investigation showed that ZnO NP-induced cell death corresponds with ferroptosis's definition. In our study, green synthesized ZnO NPs caused ferroptosis in leukemia cells by disrupting redox homeostasis, decreasing the levels of TAC with elevated 10.13039/100007293TOS and intracellular iron levels, which was further supported by significantly elevating *ACSL4*, *ALOX15*, and decreasing *SLC7A11* and *GPx4* mRNA expressions. However, this study still has limitations, including the absence of in vivo testing using mouse models, which limits the ability to observe the NP's effects on living organisms and their systemic effects and toxicity. Additionally, the study does not involve protein analysis, which restricts understanding molecular mechanisms and specific proteins involved in ferroptosis-based anti-leukemic activities.

Furthermore, it does not explore other pathways like autophagy, limiting the comprehensive knowledge of biological processes influenced by ZnO NPs. Thus, Future research could explore the systemic effects, biodistribution, and potential toxicity of ZnO NPs using mouse models. Protein analysis and other potential pathways could help understand the molecular mechanisms behind their anti-leukemic activities. Techniques like proteomics or Western blotting could be used for protein analysis. Understanding why some cells may develop resistance to ZnO NP-induced ferroptosis could be another area of study. Also, combining ZnO NPs with other anti-leukemic drugs could lead to more effective therapies, further validating the observed effects.

## 5 Ethics approval and consent to participate

The study gained the approval of the ethical committee of the Kerman University of Medical Sciences with the Ethical approval code: IR.KMU.REC.1400.371. Written informed consent was obtained from all participants. All methods were performed following the relevant guidelines and regulations.

## Consent for publication

Not applicable.

## Availability of data and materials

The data would be available from the corresponding author upon reasonable request.

## Funding

This study was supported by grant No. 99000213 from the 10.13039/501100004621Kerman University of Medical Sciences.

## CRediT authorship contribution statement

**Muhammad Hossein Ashoub:** Writing – review & editing, Writing – original draft, Software, Methodology, Investigation. **Mahnaz Amiri:** Writing – review & editing, Visualization, Validation, Supervision, Resources, Project administration, Funding acquisition, Formal analysis. **Ahmad Fatemi:** Writing – review & editing, Software, Resources. **Alireza Farsinejad:** Writing – review & editing, Visualization, Validation, Supervision, Resources, Project administration, Funding acquisition, Formal analysis, Data curation, Conceptualization.

## Declaration of competing interest

The authors declare that they have no known competing financial interests or personal relationships that could have appeared to influence the work reported in this paper.
